# The effect of cooling rate and content of niobium on the structure, wear and corrosion resistance of CoCrFeNiNb_x_ high entropy alloys

**DOI:** 10.1038/s41598-025-13551-w

**Published:** 2025-08-06

**Authors:** Rafał Babilas, Katarzyna Młynarek-Żak, Adrian Radoń, Tymon Warski, Mariola Kądziołka-Gaweł, Krzysztof Matus, Jakub Bicz

**Affiliations:** 1 Department of Engineering Materials and Biomaterials, Silesian University of Technology, Konarskiego 18a St, 44-100 Gliwice, Poland; 2 Department of Engineering Processes Automation and Integrated Manufacturing Systems, Silesian University of Technology, Konarskiego 18a St, 44-100 Gliwice, Poland; 3 Łukasiewicz Research Network - Institute of Non-Ferrous Metals, Sowińskiego 5 St, 44-100 Gliwice, Poland; 4 Institute of Physics, University of Silesia in Katowice, 75 Pułku Piechoty 1, 41-500 Chorzów, Poland; 5 Materials Research Laboratory, Silesian University of Technology, Konarskiego 18a St, 44-100 Gliwice, Poland

**Keywords:** High entropy alloys, Mössbauer spectroscopy, Scanning electron microscopy, Electrochemical impedance spectroscopy, Tribological properties, Metals and alloys, Scanning electron microscopy, Corrosion, Mechanical properties

## Abstract

In this work, CoCrFeNiNb_x_ (x = 0.25, 0.45 and 0.65) high entropy alloys were prepared by two different methods to determine the effect of cooling rate and the niobium content on the structure and properties of ingots and plates. The structure was investigated extensively using X-ray diffraction, scanning electron microscopy, and Mössbauer spectroscopy. The results confirmed the dual-phase structure, consisting of the FCC solid solution and the Laves phase. The increase in niobium content changed the microstructure from hypoeutectic (x = 0.25 and 0.45) to hypereutectic (x = 0.65). The high cooling rate during solidification from the liquid state enabled the formation of ultrafine eutectic structures with an average lamellae thickness of only 130 ± 9 nm in the CoCrFeNiNb_0.65_ plate. The corrosion behaviour of the alloys was studied in solutions of 3.5% NaCl and 3.5% NaCl + H_3_BO_3_. The beneficial effect of increasing the niobium content in as-cast CoCrFeNiNb_x_ alloys on the corrosion resistance was confirmed in both environments. Furthermore, the alloys solidified with a higher cooling rate exhibited a lower corrosion susceptibility in the 3.5% NaCl solution. The results of the EIS study indicated that a higher content of niobium contributed to the formation of a more stable and compact passivation layer. The hardness of the CoCrFeNiNb_x_ alloys increased with a higher niobium content, achieving the highest value of 669 HV_1_ for the CoCrFeNiNb_0.65_ plate. The increase in the cooling rate positively affected the tribological properties of the CoCrFeNiNb_x_ alloys, contributing to the decrease in the friction coefficient for the CoCrFeNiNb_0.25_ and CoCrFeNiNb_0.45_ plates.

## Introduction

A novel alloys development concept, called high entropy alloys (HEAs), has attracted a broad range of attention in recent years. In contrast to conventional alloys, usually based on one dominant element and several alloying additions, these alloys contain at least five principal elements, in concentrations between 5 and 35 at%. The increase in the number of elements is inevitably connected with the rise of the configurational entropy of the alloy, which reaches its maximum when the constituent elements are in equimolar ratios. It was assumed that, since HEAs can be defined as alloys with a configurational entropy exceeding 1.5 R (where R represents the gas constant)^1,2^. The high configurational entropy exhibited by these alloys contributes to the improvement in mutual solid solubility of constituent elements, promoting the formation of solid solutions while hindering emergence of intermetallic phases. In effect, HEAs often exhibit simple microstructures, in contradiction to initial predictions, which expected the formation of a brittle and complex phase structure, limiting their potential applications^1^.

HEAs exhibit various of exceptional properties, including long-term fatigue resistance, superior high-temperature microstructural stability, and high oxidation and corrosion resistance^3–7^. Consequently, these alloys are promising materials for high-temperature structural applications such as gas turbine components, combustion chambers and high-temperature moulds^3,6,8^. Furthermore, as the HEAs can exhibit improved irradiation resistance, these alloys can meet the demand for high-performance structural materials developed for next generation nuclear reactors^9–12^.

The type of structure is the dominant factor influencing the mechanical properties of high entropy alloys. The alloys with single-phase face-centred cubic (FCC) structure exhibit high ductility and relatively low strength properties. In contrast, those with single-phase body-centered cubic (BCC) structure possess high strength, however simultaneously with limited plasticity^6^. An effective way to improve the mechanical properties of alloys with a single-phase FCC structure, such as CoCrFeNi, is precipitation strengthening, which can be induced by the addition of elements with a large atomic radius, such as Mo, Nb, Ti, and Ta^13,14^. However, secondary phase strengthening is usually accompanied by a decrease in ductility. For example, the addition of Nb to the equimolar CoCrCuFeNi alloy results in the formation of intermetallic Laves phases, which enable an increase in compressive strength from 338 to 1322 MPa, simultaneously contributing to a significant loss of ductility – the fracture strain decreased from 60 to 8%^15^.

The continuous development of high-entropy alloys to reach a proper balance between strength and ductility led to the development of eutectic high-entropy alloys. The first eutectic high-entropy alloy reported was AlCoCrFeNi_2.1_, characterized by a lamellar eutectic microstructure, composed of alternating hard BCC and soft FCC phases^16^. Simultaneously with advantageous strength properties, these alloys exhibit good softening resistance and improved castability. An important feature is the possibility of obtaining an ultrafine microstructure, with a nanometric size lamella, through standard casting methods, which is attributed to the sluggish diffusion effect^17^. Advantageous properties were shown by the CoCrFeNiNb_0.5_ eutectic alloy. The composite microstructure comprised of fine and homogeneously dispersed precipitates of the Laves phase in the FCC matrix allowed for obtaining exceptional compressive strength values exceeding 2000 MPa, while sustaining fracture strain at a reasonable 17%^18^. Furthermore, it exhibits high microstructural stability at elevated temperatures, which contributes to retaining properties of relatively high strength up to 800℃, making it a promising structural material in high-temperature applications^19^.

One of the most promising research areas, associated with high entropy alloys, is the development of corrosion-resistant materials. In this field, HEAs can outperform conventional alloys, such as ferritic and austenitic stainless steel or nickel-based superalloys. In particular, superior performance shows alloys containing passivating elements, such as Cr, Ni, Al, Ti, or Nb. The corrosion resistance of alloys depends primarily on the ability to form a stable and dense passive film on their surface, preventing further corrosion^20,21^. The addition of niobium can enhance the corrosion resistance, improving the surface protection abilities of the passivation layer. It has been reported that niobium significantly affects passive film formation, promoting the generation of more Cr_2_O_3_ at the expense of Cr(OH)_3_. This effect, synergistically with the formation of Nb_2_O_5_, contributes to obtaining a denser, more stable oxide layer. However, in the case of alloys with higher niobium content, segregation caused by the formation of Laves phase contributed to a decrease in corrosion resistance^22^.

Although the influence of concentration of the niobium on the mechanical properties and corrosion resistance of CoCrFeNiNb_x_ alloys was previously well described in the literature, research on the effect of processing conditions, such as cooling rate during solidification from the liquid state – on their corrosion resistance is relatively limited. Accordingly, this work aims to investigate the structure and properties of the CoCrFeNiNb_x_ alloy (where x = 0.25, 0.45 and 0.65), prepared from the liquid state with two different cooling rates. The effects of the niobium content and the cooling rate on the structure, corrosion resistance, and mechanical and tribological properties were analysed. Furthermore, considering the beneficial combination of properties exhibited by CoCrFeNiNb_x_, such as high temperature strength^20^ and excellent corrosion resistance in chloride solutions^22^ together with improved irradiation resistance characterizing CoCrFeNi-based alloys^12^ the corrosion resistance in terms of possible nuclear applications was studied using sodium chloride and boric acid solution. The boron acid solution is used as a reactivity control agent in pressurised water reactors, as well as in the cooling pools for spent nuclear fuel rods, as boron exhibits high neutron absorption^23–25^.

## Materials and methods

The investigated HEAs were prepared in the form of ingots and plates, using high-purity elements (99.99 wt%). Alloys in the as-cast state were obtained by the induction melting with the use of a NG-40 induction generator, under an argon atmosphere. The ingots were solidified with a low cooling rate using Al_2_O_3_ crucibles. Plates with a 1 mm thickness were prepared by remelting the obtained ingots and pressure (0.06 MPa) casting using a water-cooled copper mould (with a cooling rate ~ 10^3^ K/s)^26,27^.

The phase composition of the alloys was determined using the Rigaku MiniFlex 600 X-ray diffractometer, equipped with a copper tube (Cu Kα, λ = 0.15406 nm) and a D/TEX strip detector. The microstructure of ingots and plates was analysed using the Phenom ProX scanning electron microscope (SEM), while for the extended observations, the Zeiss Supra 35 high-resolution SEM was utilized. The chemical element maps and point analyses were performed using energy-dispersive X-ray spectroscopy (EDX). Differential thermal analysis (DTA) of ingots was provided to determine the crystallization mechanism using a NETZSCH Jupiter STA 449 F3 thermal analyser. The DTA curves were recorded at 10 °C/min for heating and cooling, under a protective argon atmosphere.

The ^57^Fe Mössbauer transmission spectra were recorded at room temperature using an Integrated Mössbauer Spectroscopy Measurement System (designed by Wacław Musiał and Jacek Marzec) and a linear arrangement of a ^57^Co: Rh source, a multichannel analyser, an absorber, and a detector. The spectrometer was calibrated at room temperature with a 30 μm thick α-Fe foil. Numerical analysis of Mössbauer spectra was performed using the MossWinn4.0i programme. The Mössbauer spectroscopy method is a local study technique in which the Fe nucleus is treated as a probe of its local surroundings and can provide quantitative information concerning iron atoms in samples^28,29^. The interactions of the probe nucleus with its local environment (hyperfine interactions) provide information on the local atomic structure, lattice deformations and defects, iron oxidation states, the local microenvironments, the iron magnetic states, the relative fractions of iron-bearing components. The number of components in the Mössbauer spectrum is synonymous with the number of local Fe environments in the investigated sample.

To evaluate the corrosion resistance of the alloys investigated, electrochemical measurements were performed, in the environments of 3.5% sodium chloride solution with and without the addition of boric acid at a concentration of 2500 ppm of boron, at a temperature of 40 °C. The chemical composition of the corrosive environment with boric acid addition was selected according to publications^23,30^. Kaneko et al.^30^ justified the experiments in the NaCl-H_3_BO_3_ environment by using seawater with boric acid to cool and eliminate critical conditions in the reactor pressure vessel. The light water (not containing chlorine) is used under normal conditions, however the need to test materials for reactor applications also in terms of the possibility of occurrence of critical conditions is justified. The Autolab 302 N potentiostat, equipped with a three-electrode measuring system, was used for the study. The instrument was controlled using NOVA 1.11 software. A saturated calomel electrode (SCE) was used as a reference electrode, platinum wire was used as a counter electrode and the material was tested as a working electrode. For both environments changes in the open-circuit potential (*E*_*OCP*_) were recorded at first. Subsequently, in the case of a 3.5% NaCl solution, electrochemical impedance spectroscopy measurements were performed at open circuit potentials. The impedance data were collected over the frequency range of 10^–2^−10^5^ Hz, using perturbation signal with an AC amplitude of 5 mV. Polarization measurements were conducted in both corrosion environments, in the range of − 400 mV to 400 mV, with a scan rate of 1 mV/s. Using the Tafel extrapolation method, the corrosion potential (*E*_corr_) and the corrosion current density (*j*_corr_) were also determined.

Tribological tests of the ingots and plates were performed with the pin-on-disc method using a tribometer (CSM Instruments). The radius of the wear track was 1.5 mm, and the counter sample was a ball made of Al_2_O_3_ (*d* = 6 mm). The linear speed was 0.01 m/s, and a load of 10 N was applied. The observations of the wear tracks’ surface morphology were conducted using a Zeiss Supra 35 SEM scanning electron microscope. Hardness measurements were performed using the Future Tech FM-700 Vickers hardness instrument under a load of 1000 g for a 15 s dwell time.

## Results and discussion

**Fig. 1:**
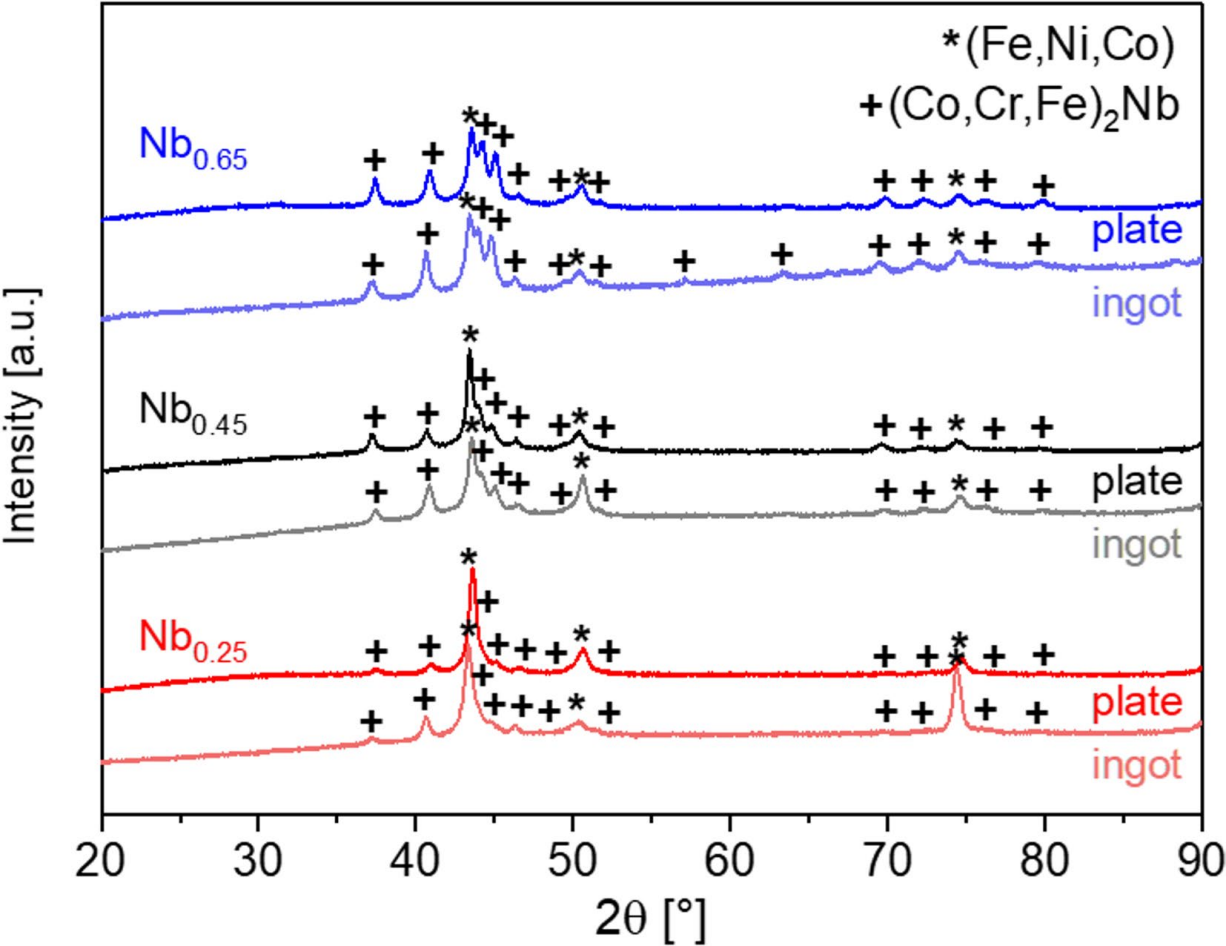
XRD patterns for the CoCrFeNiNb_x_ (x = 0.25, 0.45 and 0.65) alloys in the form of ingots and plates.

Figure 1 shows the X-ray diffraction (XRD) patterns of the CoCrFeNiNb_x_ alloys solidified with different cooling rates. All of the alloys are characterized by the dual-phase microstructure consisting of face-centred cubic (FCC) solid solution and intermetallic Laves phase, which can be identified as Co_2_Nb-type with a hexagonal close-packed (HCP) structure. As the niobium concentration increases, the diffraction peaks corresponding to the Laves phase become more pronounced, while the intensity of those corresponding to the solid solution decreases. It can be associated with a rising volume content of the intermetallic phases. Furthermore, the higher niobium content contributed to the appearance of two additional peaks correlated with the Laves phase in the case of the XRD pattern recorded for the as-cast CoCrFeNiNb_0.65_ alloy. Changes generated by the cooling rate variation can be observed: diffraction peaks corresponding to rapidly solidified alloys broadened, especially in the case of CoCrFeNiNb_0.45_ and CoCrFeNiNb_0.65_ plates. According to ref.^31^ the broadening of the diffraction peaks can be attributed to microstructure fragmentation.

**Fig. 2:**
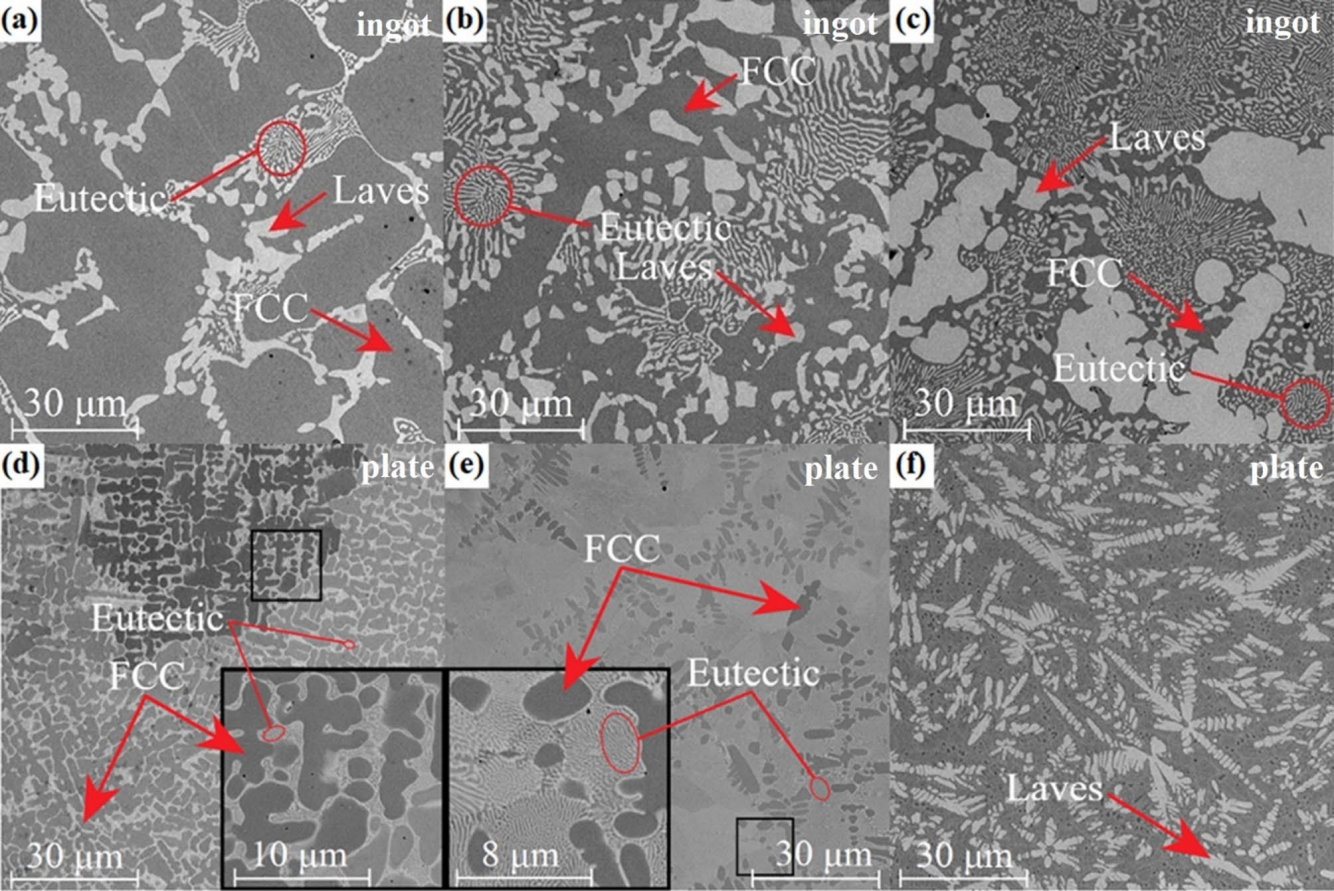
Microstructures of the CoCrFeNiNb_0.25_ (a, d), CoCrFeNiNb_0.45_ (b, e) and CoCrFeNiNb_0.65_ (c, f) alloys in the form of ingots (a-c) and plates (d-f).

**Fig. 3:**
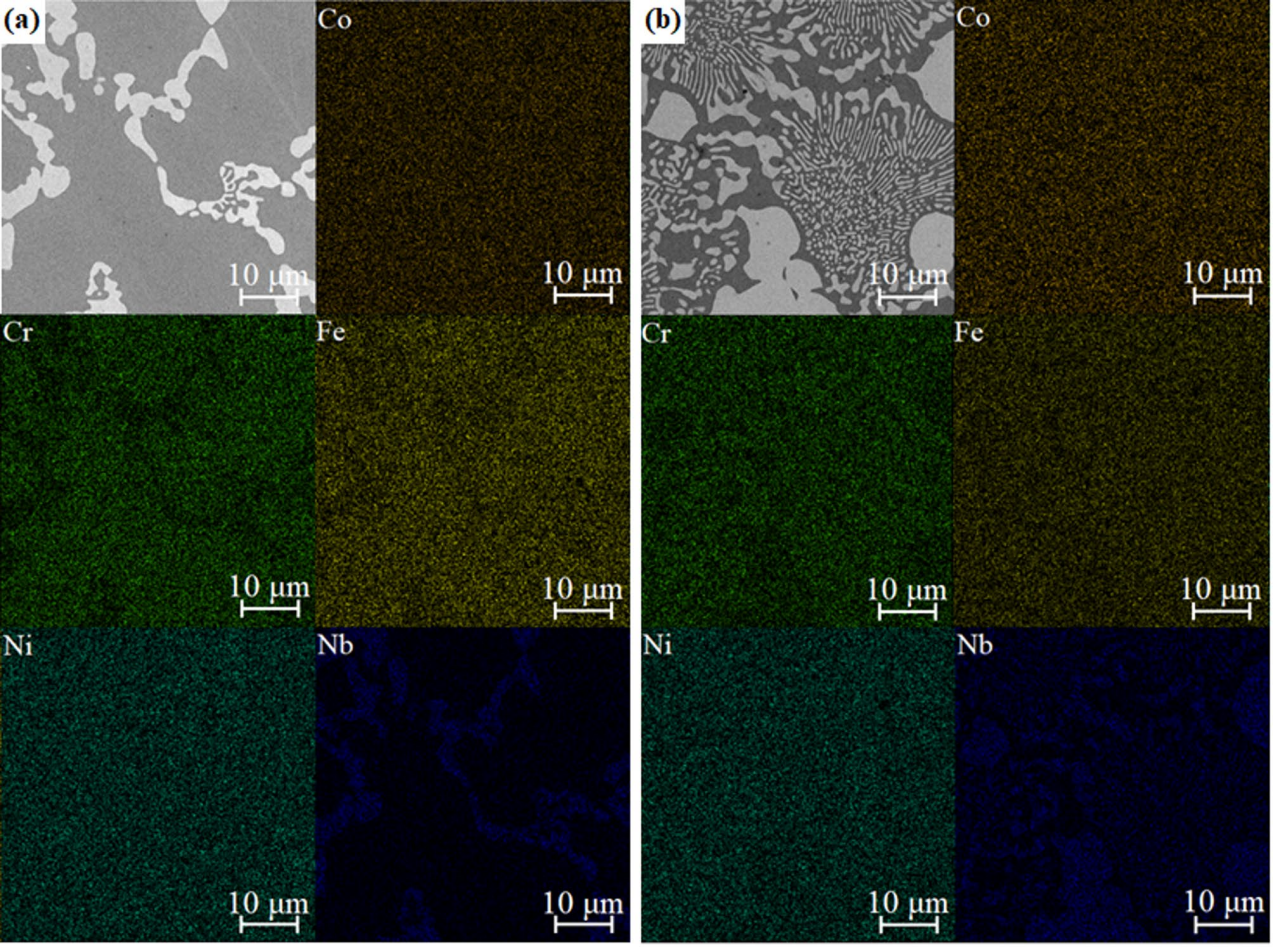
Microstructure image and results of EDX elemental distribution mapping for the CoCrFeNiNb_0.25_ (a) and CoCrFeNiNb_0.65_ (b) alloys in the form of ingots.

**Fig. 4:**
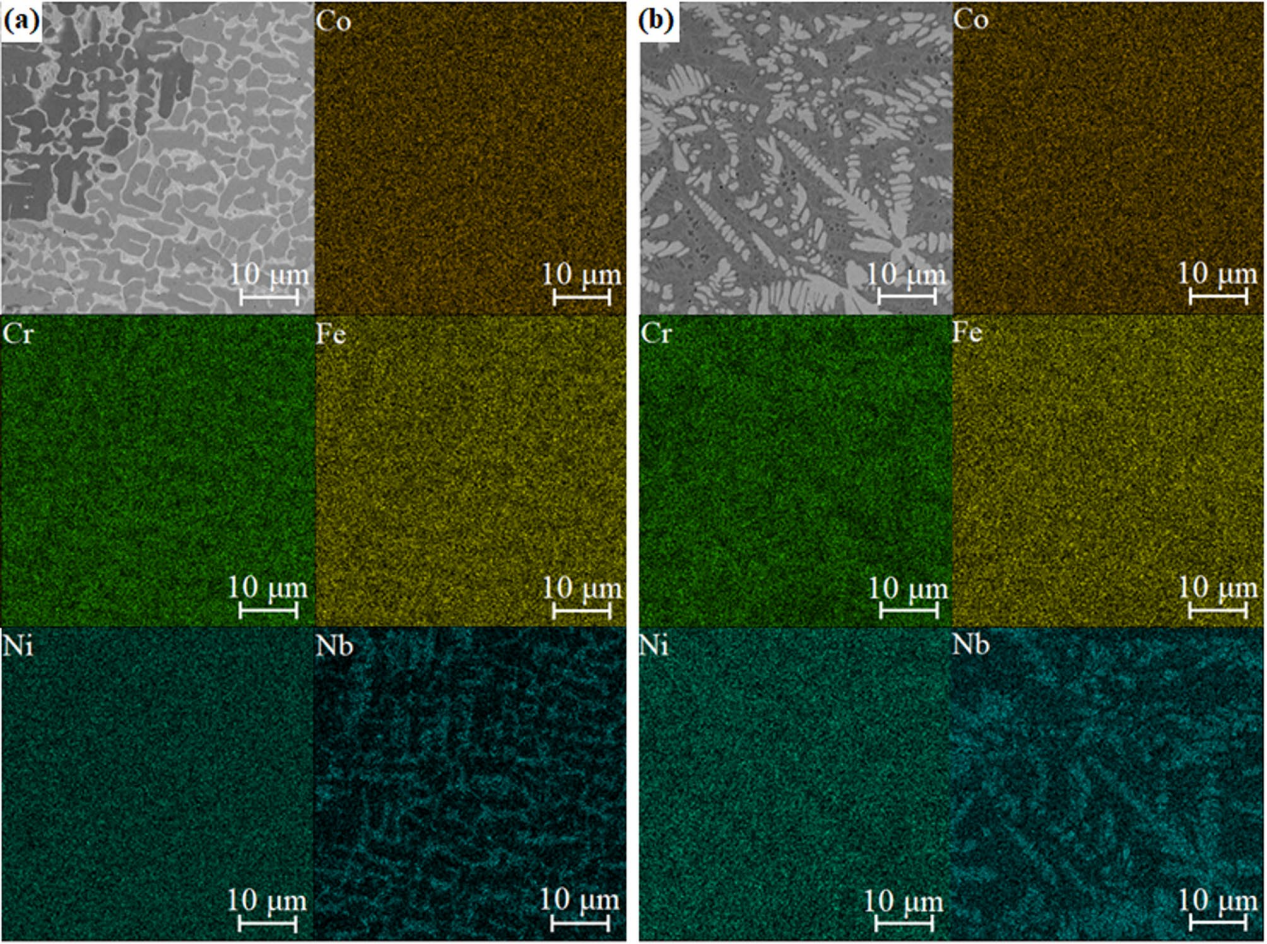
Microstructure image and results of EDX elemental distribution mapping for the CoCrFeNiNb_0.25_ (a) and CoCrFeNiNb_0.65_ (b) alloys in the form of plates.

The effect of varying the content of niobium on the microstructure of the studied alloys is seen (Fig. 2). In the case of the CoCrFeNiNb_0.25_ alloy (Fig. 2a), the microstructure is mainly comprised of dark dendrites embedded in a brighter matrix. Substantial microstructure refinement can be noticed for samples solidified with a higher cooling rate (Figs. 2d and 2f), which confirms the findings based on the results of XRD analysis. EDX point analyses were performed to determine the distribution of phases in the alloys. Subsequently, the elemental distribution maps were collected (Figs. 3 and 4) for samples in the form of ingots and plates. Table 1 summarizes the results of EDX point analyses for different regions in the microstructure of the alloys solidified with varying cooling rates.

In the case of the ingots, both phases show nearly uniform distribution of Co and Ni. At the same time, segregation can be noticed for chromium, iron, and especially for niobium (Fig. 3a). The same phenomenon appears in the case of alloy solidified with higher cooling rate (Fig. 3b). The results indicate that dark dendrites are depleted of niobium and thus can be identified as the FCC solid solution with limited niobium solid solubility. Bright precipitates can be further identified as the Laves phase, enriched with niobium and depleted of iron and chromium. Accordingly, the image analysis was performed to determine the changes in the concentration of the Laves phase caused by the increasing niobium addition into the alloy. The analysis results are presented in Table 2. As can be seen for alloys in the as-cast state as well as in the form of plates, the concentration of the intermetallic phase increases from 20.5 ± 3.4% for CoCrFeNiNb_0.25_ to 48.3 ± 2.8% for CoCrFeNiNb_0.65_, confirming the results from the XRD analysis. Similar changes were observed for the alloys solidified with a higher cooling rate. However, the fragmentation of the structure and formation of fine lamellar microstructure, also visible in Fig. 2, make it difficult to determine the phase composition in these samples precisely. Therefore, the slightly higher concentration of Laves phases in these samples should not be connected with the higher cooling rate, but with the errors in the phase concentration determination from the image analysis.

For the CoCrFeNiNb_0.25_ alloy in the as-cast state, two different types of structure can be seen in the interdendritic regions (Fig. 2a): larger, bright Laves phase precipitates and finer lamellar eutectic structures. A higher magnification image, presented in the inset of Fig. 2d, shows the presence of lamellar eutectic in an interdendritic matrix of the rapidly solidified alloy.

The higher concentration of niobium, in the case of the CoCrFeNiNb_0.45_ alloy, contributed to an increase in the content of eutectic structures, resulting in a noticeable refinement of the microstructure (Figs. 2b and 2e). The microstructure comprises of eutectic colonies, dendrites constituted of the FCC solid solution, and Laves phase precipitates, which can be observed for the alloy in the as-cast state (Fig. 2b). Eutectic colonies are characterized by a spherical shape with thinner inner lamellae and thicker outer lamellae. Radial growth and thickening of the eutectic lamellae are recognised as a specific feature of high entropy alloys^17^. In the case of the rapidly solidified alloy, a more homogeneous structure can be observed, comprised primarily of lamellar eutectic, with a smaller amount of FCC dendrites (Fig. 2e). Eutectic colonies of elongated shape are distinguishable, and no evident lamellae coarsening toward grain boundaries can be noticed.

In the case of CoCrFeNiNb_0.65_, the microstructure is composed of brighter dendrites (Figs. 2c and 2f), surrounded by eutectic colonies and smaller regions of the FCC solid solution. For the as-cast ingot, a further increase in volume content of eutectic structures can be observed, whereas in the case of the rapidly solidified plate, a decrease in favour of dendrites is observed. The alloy in plate form also had a more homogenous distribution of dendrites and was more fragmented, which can be attributed to larger undercooling and more nucleating sites than the as-cast alloy.

**Table 1:** Chemical composition of the different regions in the microstructure of the CoCrFeNiNb_x_ alloys in the form of ingots and plates based on the results of EDX point analysis.

Sample	Region	Content [at%]				
		Co	Cr	Fe	Ni	Nb
CoCrFeNiNb_0.25_ ingot	Dendrite	24.8	23.5	21.9	28.0	1.8
	Interdendritic (precipitates)	23.1	12.0	16.0	24.6	24.4
CoCrFeNiNb_0.25_ plate	Dendrite	23.9	24.9	24.9	23.0	3.4
	Interdendritic (precipitates)	21.4	20.8	19.5	21.2	17.2
CoCrFeNiNb_0.45_ ingot	Dendrite	23.0	23.4	21.5	27.6	4.5
	Interdendritic (precipitates)	23.6	12.6	14.5	23.9	25.5
CoCrFeNiNb_0.45_ plate	Dendrite	22.2	25.5	25.2	22.4	4.6
	Interdendritic (precipitates)	20.3	16.9	16.7	19.6	26.6
CoCrFeNiNb_0.65_ ingot	Dendrite	22.2	13.6	16.2	22.7	25.4
	Interdendritic (matrix)	22.7	22.1	20.7	30.5	4.0
CoCrFeNiNb_0.65_ plate	Dendrite	22.0	16.1	17.84	15.3	28.8
	Interdendritic (eutectic)	23.0	22.5	22.3	22.4	9.8

**Table 2:** Phase composition of microstructure of the CoCrFeNiNb_x_ alloys in the form of ingots and plates based on the results of SEM micrographs analysis.

Sample	Phase	Phase concentration [%]
		(mean ± SD)
CoCrFeNiNb_0.25_ ingot	FCC	79.5 ± 3.4
	Laves phase	20.5 ± 3.4
CoCrFeNiNb_0.25_ plate	FCC	70.6 ± 1.0
	Laves phase	29.4 ± 1.0
CoCrFeNiNb_0.45_ ingot	FCC	63.9 ± 0.4
	Laves phase	36.1 ± 0.4
CoCrFeNiNb_0.45_ plate	FCC	58.2 ± 4.4
	Laves phase	41.8 ± 4.4
CoCrFeNiNb_0.65_ ingot	FCC	51.7 ± 2.8
	Laves phase	48.3 ± 2.8
CoCrFeNiNb_0.65_ plate	FCC	55.8 ± 4.4
	Laves phase	44.2 ± 4.4

**Fig. 5:**
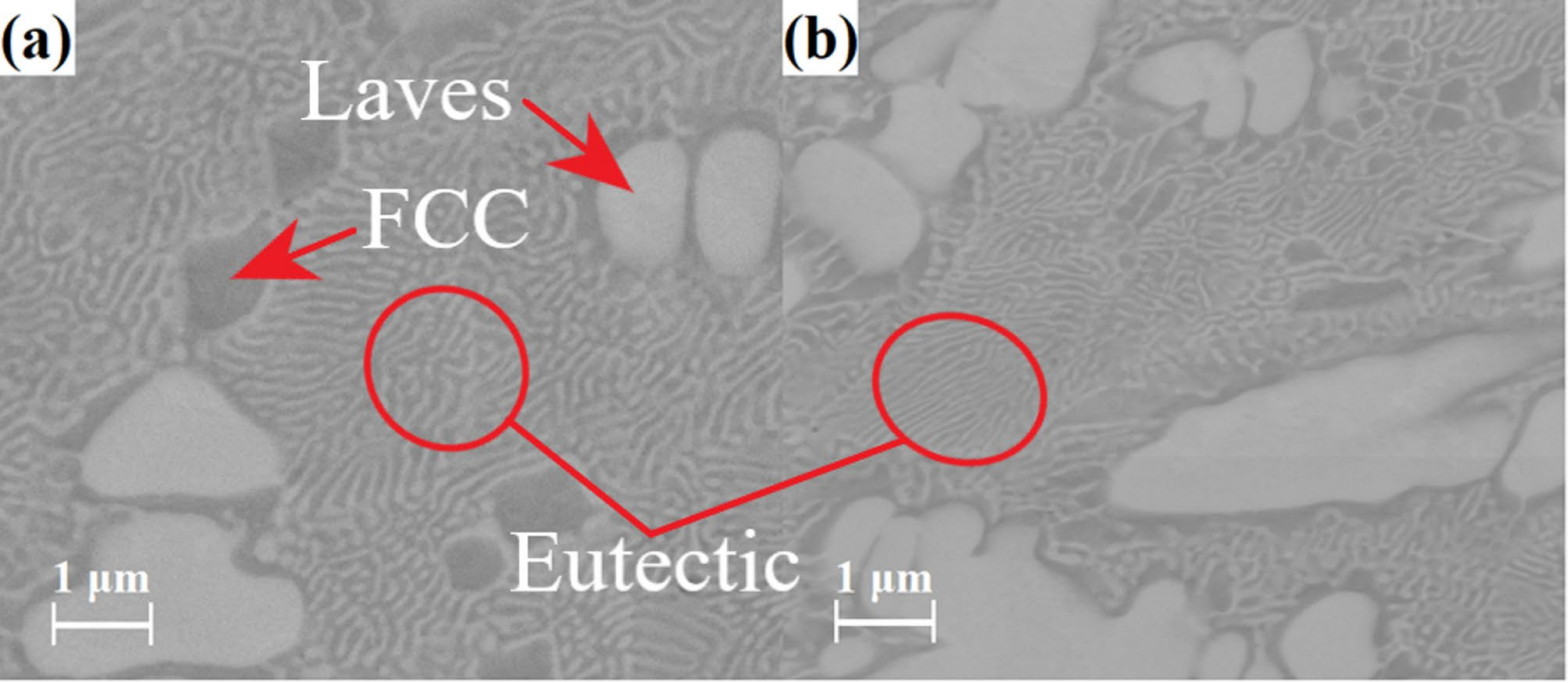
Microstructure morphology in different regions of the CoCrFeNiNb_0.65_ plate.

Simultaneously, further refinement of the eutectic structures can be noticed for samples in both forms, especially pronounced in the plate. Consequently, additional observations were made using a high-resolution scanning electron microscope to analyse the microstructure more thoroughly (Fig. 5). The average thickness of the eutectic lamellae (*λ*_w_) was measured, using the method described in ref.^32^. Based on the measurements conducted (averaged at 20 points), the *λ*_w_ parameter was estimated to be 130 ± 9 nm. The possibility of obtaining near-eutectic CoCrFeNiNb_x_ alloys with an average eutectic lamellae thickness of 200 nm and less was confirmed in earlier works^18,19^.

The EDX analysis confirmed changes in the phase distribution of the CoCrFeNiNb_0.65_ alloys. It was found that dendrites exhibit significant concentrations of niobium. Consequently, they can be identified as composed of the Laves phase (Figs. 3b and 4b). In turn, dark regions of the matrix, with lower niobium concentration, correspond to the FCC solid solution. Consequently, it can be stated that two compositions with a lower niobium content correspond to the hypoeutectic region, whereas CoCrFeNiNb_0.65_ corresponds to the hypereutectic region^18,33^. However, a significant amount of eutectic structures in the CoCrFeNiNb_0.45_ and CoCrFeNiNb_0.65_ alloys indicates that their composition is close to eutectic.

**Fig. 6:**
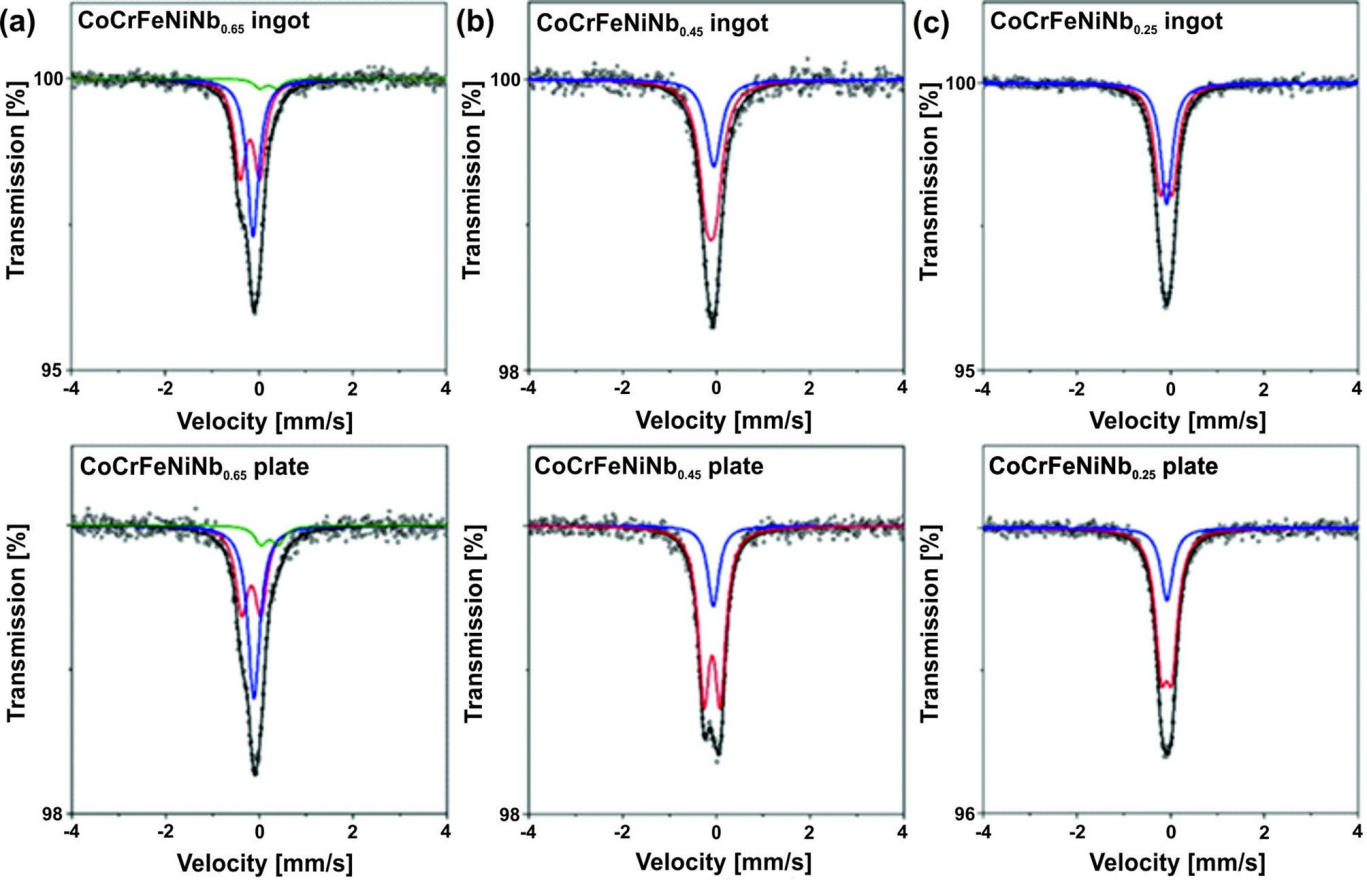
Mössbauer spectra for the CoCrFeNiNb_x_ alloys solidified with different cooling rate with x = 0.65 (a), 0.45 (b), and 0.25 (c). The experimental points, fitting curves and spectral components corresponding to particular iron sites in different phases (colored lines) are presented; red line – (Fe, Co, Cr)_2_Nb, blue line – FCC-Fe(Cr, Ni), olive line – Nb(Fe).

The Mössbauer transmission spectra of the CoCrFeNiNb_x_ alloys (x = 0.25, 0.45, and 0.65) in as-cast and plate forms are presented in Fig. 6. The hyperfine parameters for all fitted components are listed in Table 3. At room temperature, no magnetic components were observed in the spectra, only paramagnetic ones. All Mössbauer spectra were fitted with a single line and one (x = 0.25 and 0.45) or two (x = 0.65) quadrupole doublets. A single line visible in all spectra, with a negative isomer shift value (Table 2), is associated with a chromium (Cr < 14.3 at%) FCC phase based on FCC iron^34,35^ with the addition of Ni stabilizing this structure^34^. A quadrupole doublet, also with negative isomer shift values indicates, the presence of Fe in the Laves phase Fe_2_Nb^36^. The results of XRD measurements indicate the presence of this phase (Fig. 1). Slight differences in the values of hyperfine parameters obtained for this doublet compared to those observed in ref.^36^ results from the substituting Fe atoms in this structure with Co or Ni atoms^37,38^ indicating high disorder and defects. Another doublet is visible in the spectra for the CoCrFeNiNb_0.65_ alloy (Fig. 6a), both in the as-cast and plate forms. The hyperfine parameters of this doublet (Table 3) are significantly different from those characterizing the structures described above. This component is probably related to the precipitation of Nb in this alloy. If the component associated with such a phase is visible in Mössbauer spectra, this phase must be doped with Fe.

**Fig. 7:**
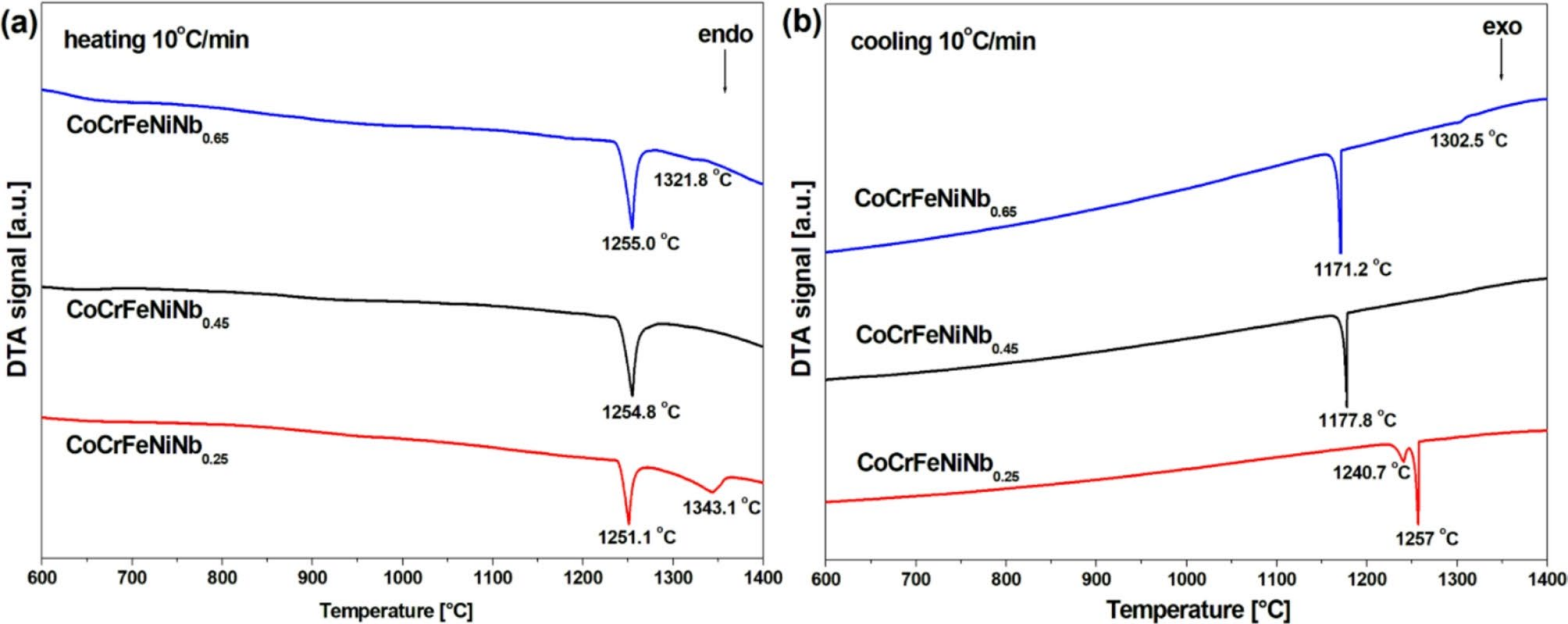
DTA curves of the CoCrFeNiNb_x_ HEA for ingots after heating (a) and cooling (b) at 20 °C/min.

DTA measurements of heating and cooling processes were conducted for samples in the as-cast state (Fig. 7). In the case of the heating curve recorded for the CoCrFeNiNb_0.25_ alloy, two thermal events were recorded – a distinct peak at 1251.1 °C and a less pronounced thermal event at 1343.1 °C. During cooling, a double heat event can be noticed consisting of a low enthalpy event at 1240.7 °C, and a more pronounced peak at 1257 °C. Moreover, the heating curve of the CoCrFeNiNb_0.45_ alloy shows the presence of a single endothermic peak, recorded at a temperature of 1254.8 °C. Correspondingly, one thermal event was recorded during cooling, at a temperature of at 1177.8 °C. For the CoCrFeNiNb_0.65_ alloy, two endothermic peaks can be observed on the heating curve, the thermal event with larger enthalpy at 1255 °C, followed by the less pronounced peak at 1321.8 °C. In the case of cooling the occurrence of two thermal events was recorded – the pronounced peak at 1171.2 °C and effect with low enthalpy at 1302.5 °C.

Based on the differential thermal analysis results, Chanda et al.^32^ determined the eutectic melting point (1261 °C) and liquidus temperature (1276 °C) of CoCrFeNiNb_0.5_ eutectic alloy. In turn, published results of calorimetric tests for CoCrFeNiNb_0.45_^19^, indicate the existence of a single thermal event with the onset at 1230 °C. According to the literature^18,19,32^ the thermal events recorded during the heating of investigated alloys, at a temperature below 1250 °C, can be related to the melting of the eutectic. Simultaneously, the corresponding exothermic peaks on the cooling curves represent the solidification points of the eutectic. In the case of the alloy with the lowest niobium content, the second, less pronounced peak on the heating curve can be attributed to the dissolution of the Laves-phase precipitates. Similarly, for the CoCrFeNiNb_0.65_ alloy, the appearance of the minor peak at the 1321.8 °C can be assigned to the melting of the Laves phase. Increasing the niobium content slightly shifts of the melting peak to higher temperatures. More pronounced differences can be seen in the case of cooling curves: the high enthalpy effect occurs at a significantly lower temperature for near-eutectic alloys, as compared to the CoCrFeNiNb_0.25_ alloy.

**Table 3:** Mössbauer hyperfine parameters of the investigated CoCrFeNiNb_x_ alloys (x = 0.25, 0.45, and 0.65) in the form of ingots and plates; IS – isomer shift, QS – quadrupole splitting, G – full line width at half maximum. Estimated errors are ± 0.01 mm/s for IS and G; for QS is ± 0.02 mm/s.

Sample	Form	IS [mm/s]	QS [mm/s]	G [mm/s]	Interpretation
CoCrFeNiNb_0.25_	Ingot	−0.09	0.27	0.30	(Fe, Co, Cr)_2_Nb
		−0.08	-	0.30	FCC-Fe(Cr, Ni)
	Plate	−0.08	0.23	0.31	(Fe, Co, Cr)_2_Nb
		−0.08	-	0.31	FCC-Fe(Cr, Ni)
CoCrFeNiNb_0.45_	Ingot	−0.12	0.19	0.38	(Fe, Co, Cr)_2_Nb
		−0.06	-	0.38	FCC-Fe(Cr, Ni)
	Plate	−0.08	0.35	0.29	(Fe, Co, Cr)_2_Nb
		−0.06	-	0.29	FCC-Fe(Cr, Ni)
CoCrFeNiNb_0.65_	Ingot	−0.19	0.42	0.30	(Fe, Co, Cr)_2_Nb
		−0.12	-	0.30	FCC-Fe(Cr, Ni)
		0.20	0.36	0.30	Nb(Fe)
	Plate	−0.17	0.41	0.32	(Fe, Co, Cr)_2_Nb
		−0.12	-	0.32	FCC-Fe(Cr, Ni)
		0.22	0.37	0.32	Nb(Fe)

**Fig. 8:**
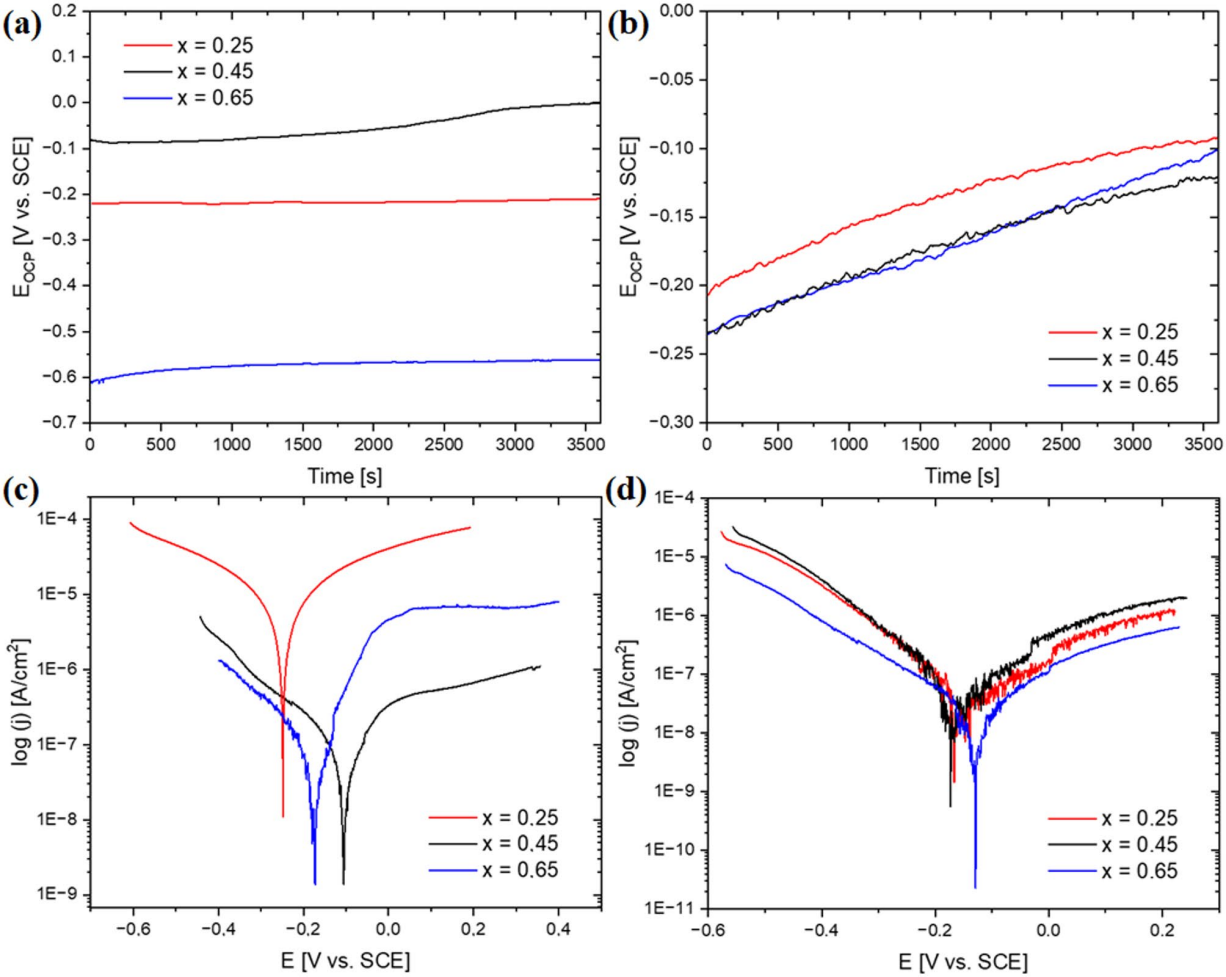
Open circuit potential as a function of time (a, b) and potentiodynamic polarization curves (c, d) for samples in the form of ingots and plates measured in a 3.5% NaCl solution at 40℃.

Electrochemical measurements using the potentiodynamic method in 3.5% NaCl solution at 40 °C were carried out to characterize the effect of niobium content and solidification rate on the corrosion resistance. Figures 8a and 8b present the changes in open-circuit potential as a function of time, whereas Figs. 8c and 8d show the recorded polarization curves. More active open-circuit potentials recorded for alloys in the form of plates, indicating growth of the passive layer, can be related to differences in surface quality. The parameters obtained in the result of conducted measurements, for samples in the form of ingots and plates, were summarized in Table 4.

A clear trend in alloys in as-cast state can be seen: an increase in niobium content contributes to a significant decrease in corrosion current density. Furthermore, for two alloys with higher niobium content, the shift in corrosion potential toward more positive values can be noticed, as well as the increase in polarization resistance by two orders of magnitude, evidencing improved corrosion resistance. On the contrary, in the case of alloys solidified with a higher cooling rate, the obtained corrosion current density values were practically comparable, which can be attributed to a more homogeneous microstructure. However, a positive shift in corrosion potential for CoCrFeNiNb_0.65_ alloy can be noticed, simultaneously with a significant increase in polarization resistance, which indicates its lower corrosion susceptibility. The positive effect of a higher cooling rate from the liquid state on the corrosion resistance can be observed for all investigated alloy compositions, which is evidenced by lower corrosion current density and higher polarization resistance values. In particular, significant improvement in corrosion behaviour can be observed when comparing the as-cast and rapidly solidified CoCrFeNiNb_0.25_ alloy.

**Table 4:** Results of the electrochemical tests conducted in a 3.5% NaCl solution at 40℃.

Sample	Form	E_corr_ [mV] (± 10)	j_corr_ [µA/cm^2^] (± 0.1)	*R*_*p*_ [kΩcm^2^] (± 0.1)
CoCrFeNiNb_0.25_	Ingot	− 248	8.59	3.8
	Plate	− 175	0.20	347.2
CoCrFeNiNb_0.45_	Ingot	− 105	1.32	134.0
	Plate	− 179	0.28	209.2
CoCrFeNiNb_0.65_	Ingot	− 178	0.42	134.9
	Plate	− 130	0.29	472.8

Compared with the CoCrFeNi alloy described in ref.^39^, an increase in the corrosion resistance can be observed in the case of the as-cast CoCrFeNiNb_0.25_ and CoCrFeNiNb_0.45_ alloys, although simultaneously comparable parameters were obtained for the CoCrFeNiNb_0.65_ alloy. However, applying a higher cooling rate during solidification ensures microstructure fragmentation and a more homogeneous elemental distribution, which contributes to the obtaining of noticeably more favourable parameters. Similarly to our work, the positive effect of increasing the niobium content on the corrosion resistance of the as-cast CoCrFeNiNb_x_ alloys was noticed in ref.^40^ comparing the corrosion behaviour of alloys with the hypo- and hypereutectic structure, in 3.5% NaCl solution, at a temperature of 25 °C. Liao et al.^41^. investigated the influence of the addition of niobium on the corrosion resistance of the Al_0.3_CoCrFeNiNb_x_ alloys (where x = 0.25, 0.35, 0.4, 0.45, 0.5, 0.75) prepared by arc melting and injection casting into copper moulds. The corrosion resistance of the alloys initially increases with the concentration of niobium, evidenced by the decrease in corrosion current density and a positive shift of the corrosion potential, which was attributed to the increase in the volume content of the eutectic structures. The ultrafine lamellar eutectic contributes to more homogenous distribution of niobium, which translates into higher passive film homogeneity and consequently enhanced corrosion resistance. However, as the niobium concentration exceeds the 10.11% at. (x = 0.45) the inverse trend was observed for both parameters, which can be related to the appearance of Laves phase dendrites. Similarly, in this work, microstructural refinement and homogenisation with increase in niobium concentration positively affected the corrosion resistance of the alloys. Although the results for the corrosion potential obtained in the ref.^41^ were more favourable compared to the as-cast alloys described in our work, the CoCrFeNiNb_x_ alloys simultaneously characterised with significantly lower corrosion current density values. In turn, Liu et al.^22^ investigated the dependence of corrosion resistance on the niobium content in the CoCrFeNiNb_x_ alloy (where x = 0, 0.15, 0.33, 0.5) prepared using the arc melting method in a solution of 3.5% NaCl at 25 °C. The results obtained in that study indicated that the best corrosion resistance characterizes the CoCrFeNiNb_0.15_ alloy, which exhibited the lowest corrosion current density of 4.028 µA/cm^2^ and corrosion potential of − 476 mV. A marginally less favourable result was obtained in our study for the as-cast CoCrFeNiNb_0.25_ alloy (8.59 µA/cm^2^), but at lower corrosion potential value (− 248 mV). Compared to CoCrFeNiNb_0.33_ and CoCrFeNiNb_0.5_ alloys, significantly lower corrosion current density values were obtained for the as-cast and rapidly solidified alloys described in this work. There are also more pronounced differences in the case of the corrosion potentials.

**Fig. 9:**
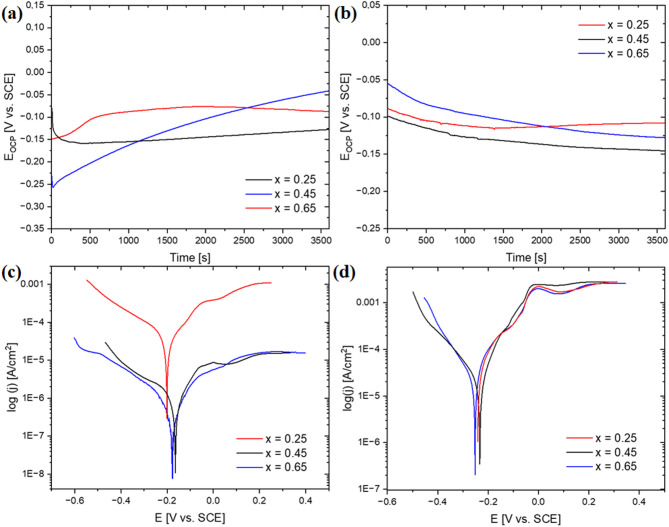
Open circuit potential as a function of time (a, b) and potentiodynamic polarization curves (c, d) for samples in the form of ingots and plates, measured in a 3.5% NaCl solution with the addition of boric acid at 2500 ppm of boron at 40℃.

Electrochemical measurements were made in the 3.5% NaCl solution with the addition of boric acid at 2500 ppm of boron at a temperature of 40 °C to evaluate further the corrosion resistance of the alloys investigated. Changes in open-circuit potential in a function of time and recorded polarization curves were shown in Figs. 9a and 9c for samples in the as-cast state, whereas for samples in the form of plates in Figs. 9b and 9d, respectively. The quantitative parameters of the corrosion potential, the corrosion current density, and the polarization resistance were summarized in Table 5. Comparison with the results obtained for tests performed in a 3.5% NaCl solution allows to determine the significant impact of the environment on the corrosion behaviour of the alloys. The corrosion current density values noticeably increase for 3.5% NaCl with the addition of H_3_BO_3_, as compared to previous measurements conducted only in 3.5% NaCl solution. An inverted tendency can be observed for polarization resistance values. Furthermore, a negative shift in corrosion potential values can be observed for alloys in the form of plates.

**Table 5:** Results of the electrochemical tests conducted in a 3.5% NaCl solution with the addition of H_3_BO_3_ at the 2500 ppm of boron at 40℃.

Sample	Form	E_corr_ [mV] (± 10)	j_corr_ [µA/cm^2^] (± 0.1)	*R*_*p*_ [kΩcm^2^] (± 0.1)
CoCrFeNiNb_0.25_	Ingot	− 201	65.3	0.49
	Plate	− 241	22.7	0.86
CoCrFeNiNb_0.45_	Ingot	− 157	5.1	7.32
	Plate	− 233	22.1	0.64
CoCrFeNiNb_0.65_	Ingot	− 178	1.6	20.4
	Plate	− 252	14.8	0.59

Similarly, as in the case of the previously studied corrosion environment, in the case of the alloys in the as-cast state, the corrosion current density decreases with increasing niobium content, indicating its positive effect on corrosion resistance. Furthermore, a substantial increase in the polarization resistance can be noticed, exceeding two orders of magnitude. The improved corrosion resistance of two alloys with a higher niobium concentration is further evidenced by the positive shift in the corrosion potential compared to the CoCrFeNiNb_0.25_ alloy. Although, in the case of CoCrFeNiNb_0.25_ alloy, the increase in cooling rate contributed to a lower corrosion current density and a higher polarization resistance, for the remaining two alloy compositions, an inverse effect was observed. For alloys in the form of plates, less favourable corrosion potential values were recorded, characterizing simultaneously with significantly lower variation. A slight decrease in corrosion current density value can be noticed for the CoCrFeNiNb_0.65_ plate, compared to rapidly solidified alloys with lower niobium content. Based on the tests conducted, it can be stated that the as-cast CoCrFeNiNb_0.65_ alloy exhibits the best corrosion resistance, indicated by the lowest value of the corrosion current density (1.6 µA/cm^2^) and the highest value of the polarization resistance (20.4 kΩcm^2^).

**Fig. 10:**
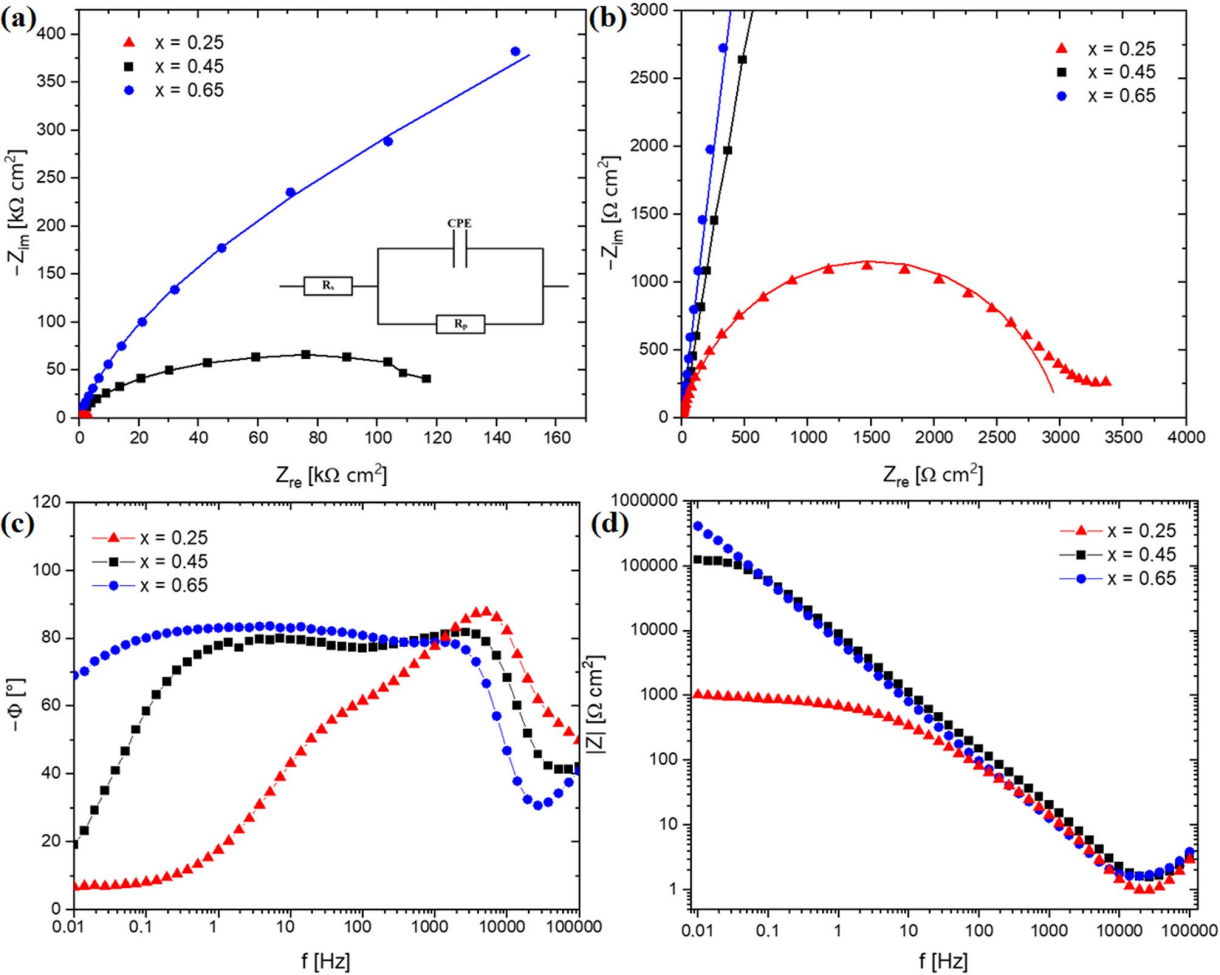
Experimental EIS spectra: Nyquist plots with solid lines representing the fitting results (a), enlarged initial section of the diagram shown at (b), Bode phase angle plots (c), Bode modulus plots (d) for the CoCrFeNiNb_x_ alloy in the form of ingots in a 3.5% NaCl solution at 40 °C.

**Fig. 11:**
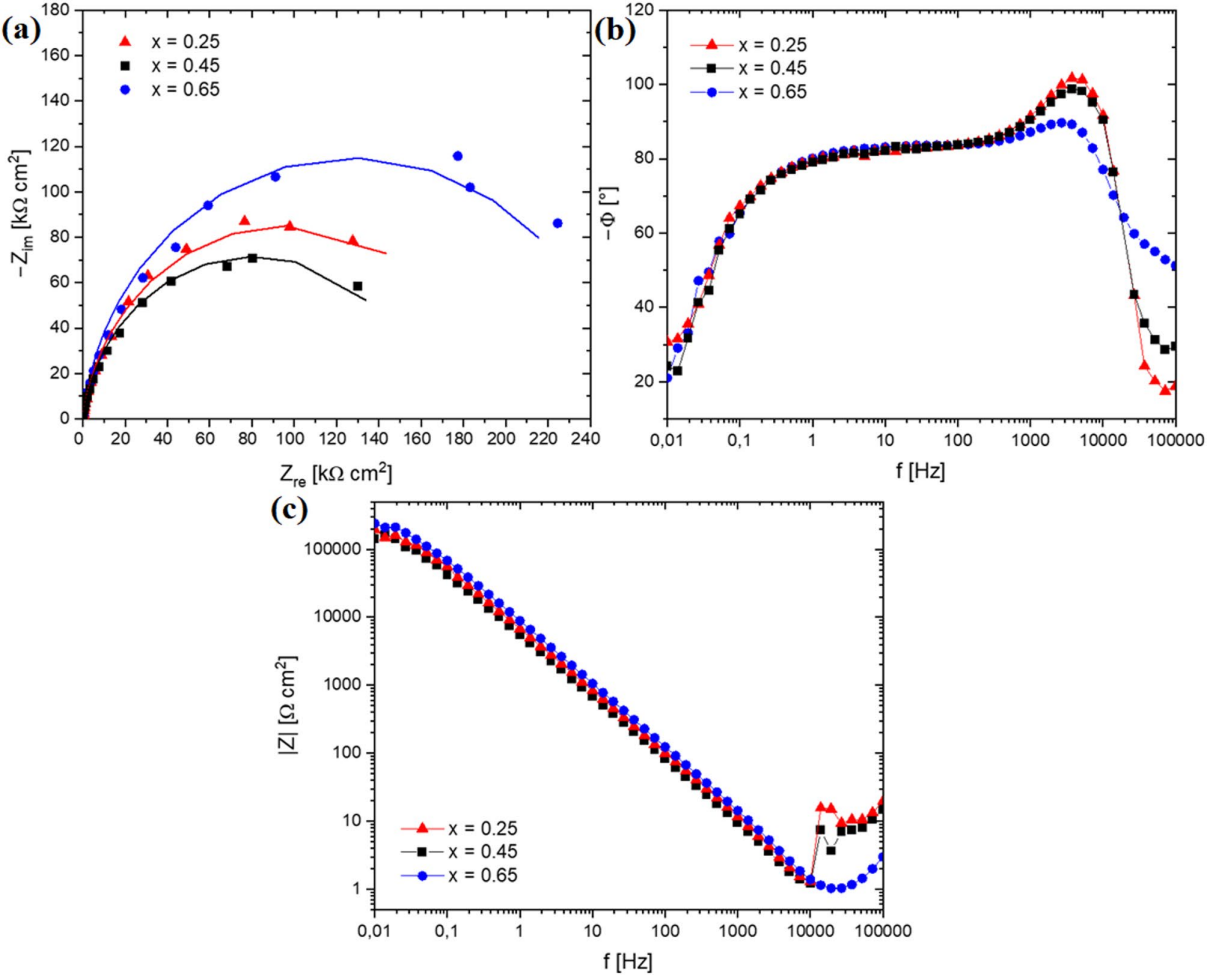
Experimental EIS spectra: Nyquist plots with solid lines representing the fitting results (a), Bode phase angle plots (b), Bode modulus plots (c) for the CoCrFeNiNb_x_ alloy in the form of plates in a 3.5% NaCl solution at 40 °C.

To further study the passivation behaviour of the CoCrFeNiNb_x_ alloy, electrochemical impedance spectroscopy (EIS) measurements were conducted. Figures 10 and 11 show the Nyquist and Bode plots of the samples in the form of ingots and plates, measured in a 3.5% NaCl solution at 40 ℃. Nyquist plots recorded for as-cast and rapidly solidified alloys (Figs. 10a and 10b and 11a) are characterized by unfinished semicircles. According to ref.^42,43^ the semicircles in the high-frequency range are related to the charge transfer at the electrode interface, and their diameter reflects the interface resistance of the charge transfer. The larger diameter of the semicircle indicates greater stability of the passivation layer. In the case of the alloys in the as-cast state, a clear tendency can be seen that a semicircle diameter increases with the niobium content, indicating an enhancement in the corrosion resistance.

Different behaviour can be observed for the solidified samples with a higher cooling rate, although still, the highest resistance characterizes the CoCrFeNiNb_0.65_ alloy. The decrease in semicircle diameter observed for CoCrFeNiNb_0.45_ indicates a deterioration of passive layer resistance compared to the CoCrFeNiNb_0.25_ alloy. The Bode plots recorded for the CoCrFeNiNb_0.45_ and CoCrFeNiNb_0.65_ as-cast alloys (Fig. 10c) show that phase angle values close to − 80° were sustained over a wide range of frequencies, indicating the formation of a stable passive layer on the surface of the alloys^42^. A different behaviour can be observed for the alloy with the lowest niobium content, suggesting that a less protective passivation layer was being formed. For alloys solidified with a higher cooling rate (Fig. 11c), phase angles in the low-frequency range are even closer to – 90° indicating highly capacitive behaviour characteristic of passive materials^44^. Consequently, it can be stated that, in this corrosion environment, a stable passive film is formed on the surfaces of rapidly solidified alloys.

The electric equivalent circuit model used for fitting the obtained impedance spectra is shown in the inset in Figs. 10a and 11a. The circuit consists of solution resistance (*R*_s_), passive film resistance (*R*_p_), and constant phase element (CPE) representing the electrical double-layer capacitance at the electrode interface. The constant phase element was utilised instead of simple capacitance, to account for the frequency dispersion behaviour related to the inhomogeneity and roughness of the electrode surface^42^.

The impedance of the constant phase element can be calculated with the use of the following equation^42,45^:

1 $$\:\text{Z}\left(\omega\:\right)={{Z}_{0}\left(i\omega\:\right)}^{-n}$$

where: *Z*_*0*_ is the capacitance of the parameter related to the electrode capacitance [F ∙ cm^–2^ ∙ s^n–1^]; *ω* is the angular frequency (rad/s) and *n* represents the constant phase exponent. Depending on the value of *n*, CPE can correspond to various electric circuits − can be purely resistive, when *n* = 0; capacitive for *n* = 1 or represent the Warburg impedance, when *n* = 0.5 ^43,46^.

The electrochemical parameters for the as-cast and rapidly solidified alloys are summarized in Table 6. For alloys in the as-cast state, the passive film resistance (*R*_p_) increases with the niobium content confirming the tendency revealed by potentiodynamic polarization measurements. In particular, a significant rise can be observed by two orders of magnitude, when comparing the CoCrFeNiNb_0.25_ and CoCrFeNiNb_0.45_ alloys, indicating a substantial improvement in passive film protective abilities. Simultaneously, there is an increase in constant phase exponent values – reflecting that surface heterogeneity decreases, suggesting the formation of a more compact passivation layer^42,46^. According to Liu et al.^22^ adding of niobium results in the formation of more Cr_2_O_3_ at the expense of Cr(OH)_3_, enhancing the passive film compactness. Concurrently, the generation of Nb_2_O_5_ further improves the stability of the passivation layer, which synergistically with the effect mentioned enhances the passive film protective abilities. Similarly, in the ref.^41^. the positive effect of niobium addition on corrosion resistance of the Al_0.3_CoCrFeNi was attributed to the enrichment of passive films with stable Cr_2_O_3_ and Nb_2_O_5_. In turn, in case of the CoCrNi alloy described in the ref.^47^, it was found that minor niobium addition promotes the formation of metal oxides and contributes to passive film thickening.

Comparing to the as-cast alloys, the polarization resistance recorded for the plates shows significantly lower variation, although simultaneously is less favourable than in the case of CoCrFeNiNb_0.65_ ingot. A noticeably higher values can be observed for the CoCrFeNiNb_0.65_ and CoCrFeNiNb_0.65_ alloys, indicating the positive effect of a higher niobium content. The constant phase exponent is very close for all samples in the form of plates, concurrently being more favourable than those obtained for as-cast CoCrFeNiNb_0.25_ and CoCrFeNiNb_0.45_ alloys, which suggests the formation of a more compact and homogenous passivation layer. The higher passive film homogeneity can be related to the more uniform elemental distribution resulting from the microstructure fragmentation.

**Table 6:** The fitted electrochemical parameters for impedance data of the CoCrFeNiNb_x_ alloy in a 3.5% NaCl solution at 40℃.

Sample	Form	*R*_S_ [Ωcm^2^]	CPE [µΩ^−1^cm^–2^s^*n*^]	*n*_1_	*R*_*p*_ [kΩcm^2^]
CoCrFeNiNb_0.25_	Ingot	1.73	56.28	0.83	3.01
	Plate	18.06	26.82	0.92	193
CoCrFeNiNb_0.45_	Ingot	0.92	22.35	0.89	145
	Plate	12.7	32.55	0.92	162
CoCrFeNiNb_0.65_	Ingot	1.30	27.33	0.92	1500
	Plate	1.77	2.07	0.93	257

**Fig. 12:**
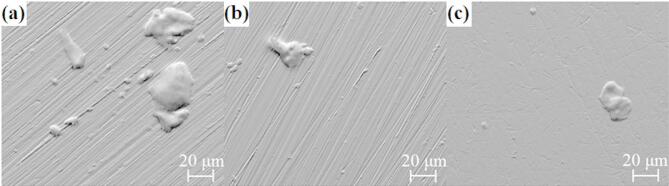
Surface morphology of samples in the form of plates after electrochemical tests in a 3.5% NaCl solution: (a) CoCrFeNiNb_0.25_, (b) CoCrFeNiNb_0.45_ and (c) CoCrFeNiNb_0.65_.

In the case of CoCrFeNiNb_x_ plates, only slight changes in surface morphology were observed after electrochemical tests. A small amount of accumulated corrosion products can be observed on their surface (Fig. 12), although simultaneously, none of the samples showed open pits. An EDX point analysis (Table 7) was performed to identify the corrosion products. Agglomerates exhibit a high oxygen concentration, simultaneously being enriched in niobium and chromium, comparing to their concentrations in the alloys, which suggests formation of a passive film with high content of niobium and chromium.

**Table 7:** Chemical composition of corrosion products after electrochemical tests in a 3.5% NaCl solution of the CoCrFeNiNb_x_ plates based on the EDX point analysis.

Sample	Content [at%]					
	Co	Cr	Fe	Ni	Nb	O
CoCrFeNiNb_0.25_ as-prepared	23.53	23.53	23.53	23.53	5.88	-
CoCrFeNiNb_0.25_ post corrosion	12.9	16.0	15.1	12.5	5.1	38.3
CoCrFeNiNb_0.45_ as-prepared	22.47	22.47	22.47	22.47	10.12	-
CoCrFeNiNb_0.45_ post corrosion	11.6	14.3	12.5	12.0	9.4	40.8
CoCrFeNiNb_0.65_ as-prepared	21.505	21.505	21.505	21.505	13.98	-
CoCrFeNiNb_0.65_ post corrosion	10.9	14.4	13.1	9.7	11.3	40.6

**Fig. 13:**
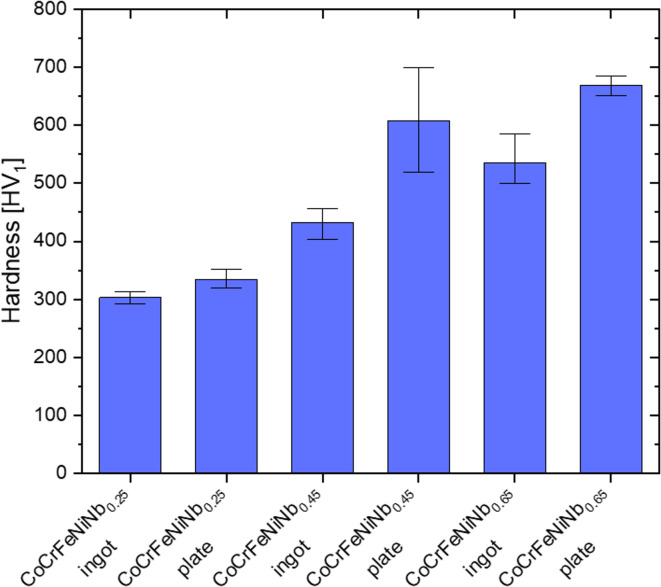
Variation of Vickers hardness of the CoCrFeNiNb_x_ alloy in the form of ingots and plates.

The results of the hardness tests of the CoCrFeNiNb_x_ alloys in the ingots and plates form are presented in Fig. 13. The beneficial effect of increasing the niobium content on the hardness of the investigated alloys is seen. The highest hardness was obtained for the CoCrFeNiNb_0.65_ alloy in the form of the plate (669 HV_1_). Comparison between the rapidly cooled (plates) and slowly cooled (ingots) samples of the same alloy indicated the positive effect of a higher cooling rate on the hardness, connected with the refinement of the microstructure. In particular, there is a substantial difference between the ingots and plates of CoCrFeNiNb_0.45_ and CoCrFeNiNb_0.65_ alloys. In the case of rapidly cooled alloys a sharp increase in hardness values can be noticed when comparing the hardness of CoCrFeNiNb_0.25_ and CoCrFeNiNb_0.45_ alloys followed by a further slight increase for the CoCrFeNiNb_0.65_ alloy.

The hardness value increased nearly linearly with niobium concentration for alloys in the as-cast state. A similar effect is observed in ref.^33^. for the CoCrFeNiNb_x_ alloy (x = 0.2, 0.4, 0.6, 1.0) prepared using the arc melting method. The hardness increased from 144 HV, obtained for the as-cast CoCrFeNi alloy, to 652 HV for equimolar CoCrFeNiNb. Sunkari et al.^14^ reported that the addition of niobium led to the rise in hardness of non-equimolar CoCrFeNi_2.1_Nb_x_ from 135 HV for the base alloy to 480 HV for CoCrFeNi_2.1_Nb_0.74_. In turn, for the eutectic alloy CoCrFeNiNb_0.5_ prepared by the arc melting method, a hardness value of 521 HV was obtained, which was attributed to the presence of a homogeneous nanolamellar microstructure^18^.

The observed positive effect of the addition of niobium on the strength properties of the CoCrFeNi alloy can be related to the increase in the volume content of the Co_2_Nb-type Laves phase (from 20.5 ± 3.4% for CoCrFeNiNb_0.25_ to 48.3 ± 2.8% for CoCrFeNiNb_0.65_) and microstructure refinement, resulting from the formation of lamellar eutectics. The Laves phase with a hexagonal close-packed structure is characterized by high hardness, contributing to a significant strengthening effect. The increase in the cooling rate also positively affects the hardness value, allowing us to obtain a more homogeneous and fragmented microstructure. Especially, in the case of the rapidly solidified alloys of CoCrFeNiNb_0.45_ and CoCrFeNiNb_0.65_, the significant volume content of the eutectic structures contributed to their higher hardness, as the ultrafine lamellar eutectic structures introduce a high density of phase boundaries, which effectively hinder the dislocation motion^32^. The most favourable hardness value was obtained for the CoCrFeNiNb_0.65_ plate, which can be attributed to the high volume content of the Laves phase and the formation of eutectic colonies with lamellae thicknesses in the nearly-nanometric range. The lowest changes in the hardness value (an increase from 303 to 334 HV_1_) were observed for the alloy with the highest volume content of the solid FCC phase solution. These changes, in turn, can be related to microstructure fragmentation.

**Fig. 14:**
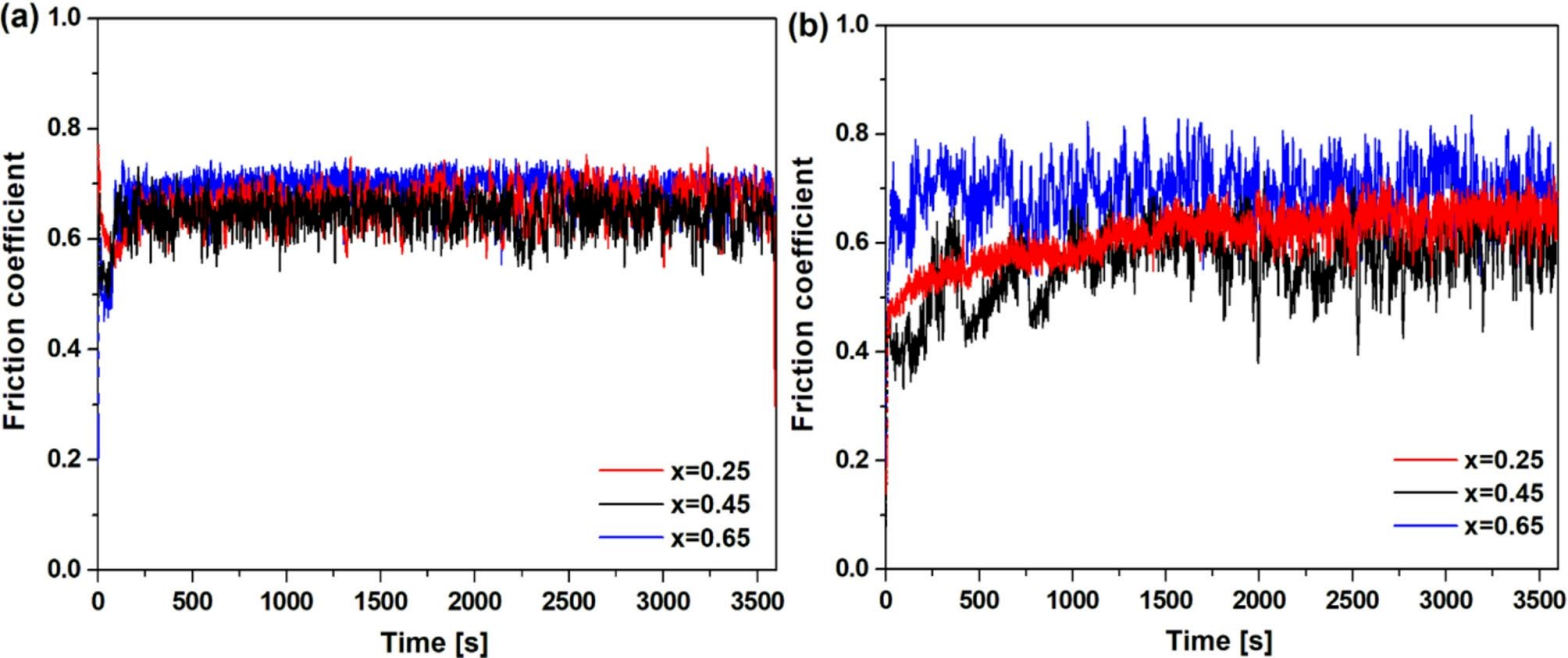
Changes of friction coefficient as function of time for the CoCrFeNiNb_x_ alloys in the form of ingots (a) and plates (b).

**Fig. 15:**
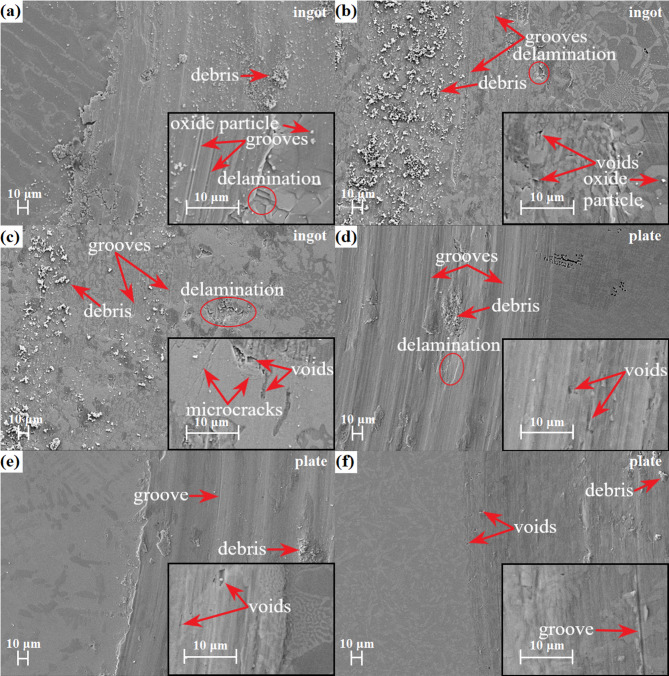
Surface morphology of the wear tracks of the CoCrFeNiNb_0.25_ (a, d), CoCrFeNiNb_0.45_ (b, e) and CoCrFeNiNb_0.65_ (c, f) alloys in the form of ingots and plates.

Tribological tests were performed using the pin-on-disc method to characterize the influence of the niobium content and the cooling rate on the wear resistance of the CoCrFeNiNb_x_ alloys. Figures 14a and 14b show the friction coefficient as a function of time, for the as-cast and rapidly solidified alloys, respectively. Figure 15 shows the wear morphologies of the as-cast and rapidly solidified CoCrFeNiNb_x_ samples. In the case of the alloys in the as-cast state a similar course of the friction curves can be observed characterizing with the increase of friction coefficient during the first 5 min of the test, followed by the subsequent stabilization (Fig. 14a). The CoCrFeNiNb_0.45_ alloy exhibited the lowest average friction coefficient of 0.64 ± 0.05, whereas for the CoCrFeNiNb_0.25_ and CoCrFeNiNb_0.65_ alloys slightly higher values of, respectively, 0.66 ± 0.07 and 0.69 ± 0.06 were obtained. The as-cast state alloys exhibited similar friction coefficients and wear mechanisms as observed in the SEM images shown in Fig. 15a-c for the pin-on-disc test samples.

**Fig. 16:**
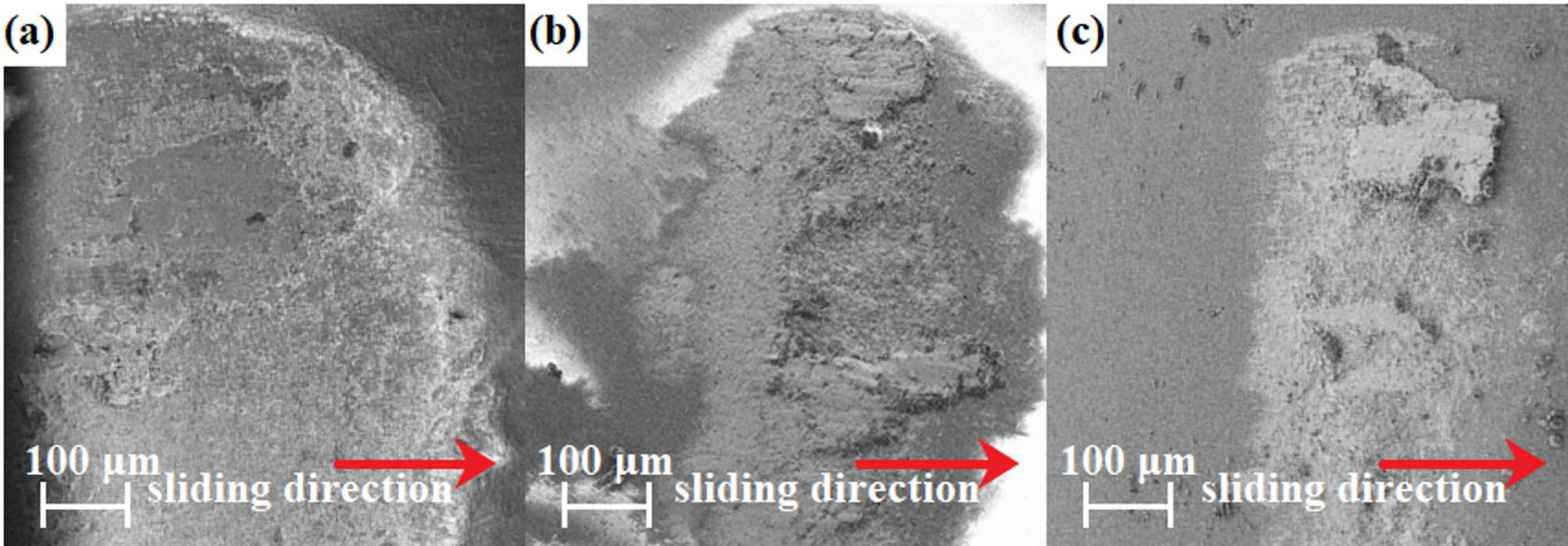
Surface morphology of the worn traces on the Al_2_O_3_ balls after sliding against the as-cast CoCrFeNiNb_0.25_ (a), CoCrFeNiNb_0.45_ (b) and CoCrFeNiNb_0.65_ (c) alloys.

Delamination can be observed for all of the alloys in the as-cast state. In addition, worn surfaces show the presence of patches of adhered material, indicative of adhesive wear. As the niobium content increases, the signs of abrasive wear become less pronounced, which can be related to an increase in hardness. More pronounced differences were shown by the rapidly solidified samples (Fig. 14b). An increase in the cooling rate contributed to a substantial decrease in the average friction coefficient in the case of the CoCrFeNiNb_0.45_ plate, allowing to reach a value of 0.56 ± 0.1. The positive effect of reducing the friction coefficient in the CoCrFeNiNb_0.45_ plate may be due to the higher proportion of the intermetallic phase in relation to the FCC phase compared to the as-cast state alloy visible on SEM images in Figs. 2b and 2e. The positive effect of a higher cooling rate on tribological properties can also be observed for CoCrFeNiNb_0.25_ alloy in a form of plate, which exhibits a slightly lower average friction coefficient value of 0.61 ± 0.08, as compared to the as-cast alloy. In turn, in the case of the CoCrFeNiNb_0.65_ plate, the average friction coefficient value of 0.69 was maintained despite the cooling rate, although in the case of the rapidly solidified alloy, a higher standard deviation (0.08) of the friction coefficient can be noticed The alloys in the form of plate also showed that with the increase of niobium, the signs of abrasive wear become less pronounced. Furthermore, the increased cooling rate contributed to the reduction of wear debris. The adhesive wear was further confirmed by the presence of a transfer film on the surface of the Al_2_O_3_ counterpart ball (Fig. 16). Similarly, grooves can also be observed on the worn alloy surface, evidencing the occurrence of abrasive wear.

It can be observed that the delamination occurs preferentially in the FCC solid solution phase. The FCC phase undergoes plastic deformation, accumulating of dislocations in the subsurface, and dislocation piles contribute to the nucleation of voids, further forming cracks that propagate parallel to the surface^48^. In turn, the high density of phase interfaces in the lamellar eutectic hinders the propagation of the crack and reduces its susceptibility to delamination^49,50^. However, simultaneously, in the case of the as-cast CoCrFeNiNb_0.45_ and CoCrFeNiNb_0.65_ alloys, the tearing marks can be observed (insets in Figs. 15b and 15c), present mainly in the solid solution phase. In the case of the latter alloy, the appearance of cracks and cavities resulting from brittle spalling can be observed in the bulk Laves phase precipitates. The loose debris, which derives from delamination and spalling, undergoes further fragmentation, trapped between sliding surfaces, generating numerous wear particles. The debris deposits on the surface are characterized by a high oxygen content (34.2 at%), which was confirmed by the conducted EDS method. During sliding, the temperature of the contact areas can locally increase to 600 °C, contributing to surface oxidation, and the chemical activity of niobium can be conducive to its selective oxidation^49,51^.

The increase in cooling rate has a noticeable effect on wear mechanisms. Delamination and abrasive wear can be observed for the rapidly solidified CoCrFeNiNb_0.25_ alloy. Moreover, the worn surface also shows the presence of adhered debris patches and tearing marks. The abrasive wear became less pronounced with increasing niobium content, which can be attributed to increased hardness. Similarly, in the case of two alloys with a higher niobium content, the amount of material adhered to the worn surfaces diminishes. Compared to alloys as-cast, delamination appears to be alleviated, which can be attributed to a more homogeneous lamellar eutectic structure characterized by higher toughness^52^. The beneficial effect of the eutectic structure on tribological properties was also shown in ref.^49^, which described the CoCrNiNb_0.385_ medium entropy alloy with a structure comprised of alternating lamellae of FCC solid solution and intermetallic Laves phase.

Joseph et al.^53^ studied the wear properties of a single-phase FCC structured Cantor alloy (CoCrFeNiMn) alloyed using a vacuum arc-melter. Pin-on-disc tests were performed with the load of 15 N and Al_2_O_3_ counter sample at various temperatures. The Cantor alloy at 25℃ showed a friction coefficient of 0.6. This value is generally similar to the results obtained in this work. However, it can be stated that the reduced value of the friction coefficient obtained for the CoCrFeNiNb_0.45_ alloy in the form of a plate may result from the obtaining of a fine eutectic in the interdendritic regions. Wang et al.^54^ studied the tribological properties of the Fe_1.87_Co_0.13_ alloy with a nanoeutectic structure and a coarse-grained structure. Despite obtaining the same friction coefficient values, according to work^54^ the wear resistance of the alloy with nanoeutetic structure was 2–6 times greater.

## Conclusions

- The CoCrFeNiNb_x_ (x = 0.25, 0.45 and 0.65) alloys are characterized by a double-phase structure, consisting of FCC solution and Co_2_Nb-type Laves phase. An increase in the niobium content caused the evolution of the microstructure from hypoeutectic, in the case of the CoCrFeNiNb_0.25_ and CoCrFeNiNb_0.45_ alloys, to hypereutectic for the CoCrFeNiNb_0.65_ alloy.
- Increasing the cooling rate contributed to the substantial refinement of the microstructure allowing to obtain ultrafine eutectic structures in the case of the rapidly solidified CoCrFeNiNb_0.65_ alloy. Based on an analysis of the Mössbauer spectra, it can be stated that all of the investigated CoCrFeNiNb_x_ alloys exhibit the presence of only paramagnetic phases at room temperature.
- Potentiodynamic measurements in 3.5% NaCl solution revealed a beneficial effect of increasing the niobium content on the corrosion resistance of alloys in the as-cast state. Different behaviours were observed during measurements in 3.5% NaCl with the addition of boric acid. Although the tendency observed in the previous environment is sustained for as-cast alloys, the positive effect of the higher cooling rate can only be observed for the CoCrFeNiNb_0.25_ alloy.
- The EIS study complemented the results of the polarization measurements. A more stable and compact passivation layer is formed with increasing niobium content for as-cast alloys in a 3.5% NaCl solution. At the same time, rapidly solidified alloys exhibit similar corrosion behaviour despite differences in the niobium content.
- The hardness of the CoCrFeNiNb_x_ alloys increased with the niobium concentration, which can attributed to the increasing concentration in the Laves phase.
- A higher cooling rate contributes to improvement of tribological properties, which was especially noticed for CoCrFeNiNb_0.45_ and CoCrFeNiNb_0.65_ plates and may be related to their very fine lamellar eutectic microstructure. The beneficial effect of an increase in the cooling rate on the friction coefficient can be observed in the case of the CoCrFeNiNb_0.25_ and CoCrFeNiNb_0.45_ alloys. Delamination, abrasive, and adhesive wear were identified as the main mechanisms.

## Data Availability

The data and material generated during and/or analyzed during the current study are available from the corresponding author upon reasonable request.
